# Clinical utility of echocardiography in bronchopulmonary dysplasia: a retrospective cohort study

**DOI:** 10.1016/j.jped.2026.101521

**Published:** 2026-03-01

**Authors:** Lie Huang, Lei Luo, Yongping Li, Jianhui Wang, Meile Cheng, Xi Xu

**Affiliations:** a The First People's Hospital of Neijiang, Department of Pediatrics, Neijiang, Sichuan, China; b Children's Hospital of Chongqing Medical University, Department of Neonatology, Chongqing, China; c The First People's Hospital of Yinchuan, Department of Neonatology, Yinchuan, Ningxia, China; d The First People's Hospital of Neijiang, Department of Science and Education, Neijiang, Sichuan, China

**Keywords:** Infants, Chronic lung disease, Cardiac function, Bronchopulmonary dysplasia, Echocardiography

## Abstract

**Objective:**

Bronchopulmonary dysplasia (BPD) is a common chronic lung complication in preterm infants, often complicated by cardiopulmonary dysfunction and poor outcomes. This study aimed to evaluate echocardiographic parameter differences at 36 weeks postmenstrual age (PMA) between preterm infants with and without BPD, and explore echocardiography’s utility for BPD identification.

**Methods:**

A retrospective cohort study included 94 preterm infants (gestational age < 32 weeks) admitted to the NICU (2019–2023). They were grouped by 2018 NICHD criteria: Non-BPD (*n* = 50, no/mild respiratory support) and BPD (*n* = 44, requiring prolonged oxygen/mechanical ventilation). Standard transthoracic echocardiography (TTE) at 36 weeks PMA assessed left ventricular (LV), right ventricular (RV), and tissue Doppler imaging (TDI) parameters.

**Results:**

Gestational age and birth weight were comparable between the two groups (*p* < 0.01), but BPD infants had longer mechanical ventilation duration (*p* < 0.01). BPD infants showed significant cardiac dysfunction: reduced LV ejection fraction (LVEF), mitral valve E velocity (MV-E), and MV-E/A ratio; decreased RV tricuspid annular plane systolic excursion (TAPSE) and fractional area change (FAC); and abnormal TDI (lower TV-S′/E′/A′, higher TV-E/E′ ratio) (all *p* < 0.01).

**Conclusions:**

Echocardiographic parameters reflecting biventricular systolic/diastolic dysfunction differ significantly between BPD and non-BPD preterm infants, indicating potential for BPD identification.

## Introduction

Bronchopulmonary dysplasia (BPD) is a chronic lung disease that predominantly affects preterm infants, with pathological features characterized by alveolar simplification and impaired pulmonary vascular development [1,2]. A 2021 multicenter study in the United States reported a 40.6 % incidence among extremely preterm infants (22–29 gestational weeks) [3], while contemporaneous Chinese data showed a 29.2 % prevalence in preterm neonates born before 32 weeks. With the advancement of perinatal technologies [[4], [5], [6]], the survival rate of extremely preterm infants has improved markedly, which has consequently made BPD one of the most prevalent complications in this vulnerable population. Notably, BPD is frequently complicated by pulmonary hypertension (pH) and cardiac dysfunction, both of which are associated with significantly increased mortality. Current research primarily focuses on identifying risk factors for BPD, optimizing respiratory management, and developing pharmaceutical treatments. Given that the pathophysiology of BPD remains unclear and specific treatments are lacking [7], early prevention and prompt intervention for this disorder should be prioritized.

Recent advancements in echocardiography technology have led to the increasing adoption of targeted neonatal echocardiography (TNE), which is performed by neonatologists in neonatal intensive care units (NICU) [8]. This diagnostic approach provides real-time hemodynamic assessment for preterm infants and offers valuable guidance for the management of critically ill newborns. Emerging evidence suggests TNE exhibits significant potential in identifying cardiovascular abnormalities in preterm infants and optimizing neonatal cardiovascular care. The development of BPD is associated with progressive structural and functional changes in the heart, and severe BPD may potentially exacerbate cardiac deterioration [9]. Clinically, TNE has been proven effective in diagnosing and evaluating various conditions, including pericardial effusion, neonatal shock, patent ductus arteriosus (PDA), and persistent pulmonary hypertension of the newborn (PPHN). However, its application in BPD research remains limited, as relatively few studies have investigated the relationship between TNE parameters in preterm infants and the development of BPD.

The authors hypothesize that preterm infants with BPD may have systemic cardiac dysfunction. This form of cardiac dysfunction may directly or indirectly increase the infants' requirements for respiratory support, thereby contributing to the development of persistent respiratory complications. The primary objective of this study is to identify differences in echocardiographic parameters between preterm infants with BPD and healthy preterm infants, with the aim of providing a foundation for further investigating the interaction between cardiac and pulmonary function in infants with BPD.

## Methods

### Study design

At our institution, serial echocardiographic screening is routinely performed at 36 (±1) weeks postmenstrual age (PMA) for all infants born at < 32 weeks of gestation. Infants were stratified into two cohorts: the BPD group (Study Group, *n* = 44) and the Non-BPD group (Control Group, *n* = 50). This retrospective study was approved by the Ethics Committee of The First People’s Hospital of Yinchuan (Approval No 2023,069). The authors analyzed data from infants admitted between January 2019 and May 2023, with the following inclusion criterion: infants born at < 32 weeks of gestation. Exclusion criteria were as follows: presence of congenital heart disease, chromosomal abnormalities, inherited metabolic disorders, need for surgical intervention, major surgical conditions, or incomplete clinical data.

### BPD definition

BPD was defined in accordance with the 2018 diagnostic criteria of the National Institute of Child Health and Human Development (NICHD) as the requirement for oxygen supplementation alone or oxygen plus respiratory support at 36 weeks postmenstrual age (PMA). The respiratory support modalities included mechanical ventilation, non-invasive positive pressure support, high-flow nasal cannula therapy, and low-flow nasal cannula therapy [10].

### Clinical definitions

To ensure diagnostic consistency, clinical variables were defined as follows:

Patent Ductus Arteriosus (PDA): Diagnosed via TTE; hemodynamic significance was defined by a ductal diameter > 1.5 mm or a left atrium-to-aortic root (LA/Ao) ratio >1.5 with left-to-right shunting.

Pulmonary Hypertension (pH): Chronic pH at 36 weeks PMA was defined by a peak tricuspid regurgitation velocity > 2.8 m/s or echocardiographic signs of RV pressure overload (e.g., septal flattening).

Necrotizing Enterocolitis (NEC): NEC was defined and staged according to the modified Bell’s staging criteria. Only cases with Stage IIa (proven NEC) or higher were included in the “NEC group.” Stage I (suspected NEC) cases were excluded from the analysis to ensure diagnostic specificity.

Intraventricular Hemorrhage (IVH): Graded according to Papile’s classification (Grade I-IV).

### Echocardiographic assessments

Comprehensive echocardiographic evaluations were carried out using a Vivid E9 ultrasound system (GE Medical Systems, Milwaukee, WI) equipped with a 4–12 MHz phased-array transducer (model: M5Sc). All examinations were performed by two certified pediatric sonographers to minimize bias. Measurements were recalculated offline by two independent investigators from stored DICOM images, and the inter-observer reliability was assessed using the Intraclass Correlation Coefficient (ICC), which yielded a value of 0.92, indicating high consistency. 

Following standardized echocardiographic protocols [[11], [12], [13]], the following cardiac parameters were assessed: left ventricular (LV) stroke volume (LVSV), LV output (LVO), LV fractional shortening (LVFS), LV end-systolic dimension (LVESD), LV end-diastolic dimension (LVEDD), LV ejection fraction (LVEF), right ventricular (RV) end-diastolic area (RVEDA), RV end-systolic area (RVESA), RV fractional area change (FAC), tricuspid annular plane systolic excursion (TAPSE), mitral valve (MV) E-wave (MV-E), MV A-wave (MV-A), MV-E/A ratio, tricuspid valve (TV) E-wave (TV-E), TV A-wave (TV-A), TV-E/A ratio, MV tissue Doppler early diastolic velocity (MV-E′), MV tissue Doppler late diastolic velocity (MV-A′), MV tissue Doppler systolic peak velocity (MV-S′), TV tissue Doppler early diastolic velocity (TV-E′), TV tissue Doppler late diastolic velocity (TV-A′), TV tissue Doppler systolic peak velocity (TV-S′), MV-E/E′ratio, and TV-E/E′ratio. LVFS was derived from M-mode tracings or two-dimensional (2D) imaging: in the parasternal long-axis (PLAX) view at the level of the mitral valve leaflet tips, or in the parasternal short-axis (PSAX) view at the papillary muscle level. LV end-diastolic dimension (LVEDD) was measured at the R-wave of the cardiac cycle, while LV end-systolic dimension (LVESD) was obtained at the end of the T-wave. LVFS was calculated using the formula: LVFS ( %) = [(LVEDD - LVESD) / LVEDD] × 100. LVEF was computed via biplane LV volume measurements from the apical four-chamber and two-chamber views, expressed as the percentage ratio of the difference between end-diastolic and end-systolic volumes to end-diastolic volume. MV-E, MV-A, and the MV-E/A ratio were detected using pulsed-wave (PW) Doppler. For RV fractional area change (FAC): RV areas at end-diastole and end-systole were quantified in the four-chamber view by tracing the endocardial borders, with RV trabeculations included within the traced area. FAC was calculated as a percentage using the formula: [(RVEDA - RVESA) / RVEDA] × 100 %. Tricuspid annular plane systolic excursion (TAPSE) was defined as the distance between the systolic and diastolic positions of the tricuspid annulus, measured in the 2D apical four-chamber view. TV-E, TV-A, and the TV-E/A ratio were detected using PW Doppler. Tissue Doppler Imaging (TDI) was employed to measure mitral and tricuspid annular velocities: the PW Doppler cursor was positioned at the mitral annulus to detect MV-E′ (early diastolic), MV-A′ (late diastolic), and MV-S′ (systolic peak); similarly, the cursor was placed at the tricuspid annulus to record TV-E′(early diastolic), TV-A′(late diastolic), and TV-S′(systolic peak) (Table S1).

### Statistical analysis

All measured data are expressed as mean ± standard deviation (SD). Intergroup comparisons were conducted using Student's *t*-tests for normally distributed variables and Kruskal-Wallis tests for non-normally distributed variables, with statistical significance defined as *p* < 0.05.

## Results

### General characteristics of the study cohort

**Figure 1:**
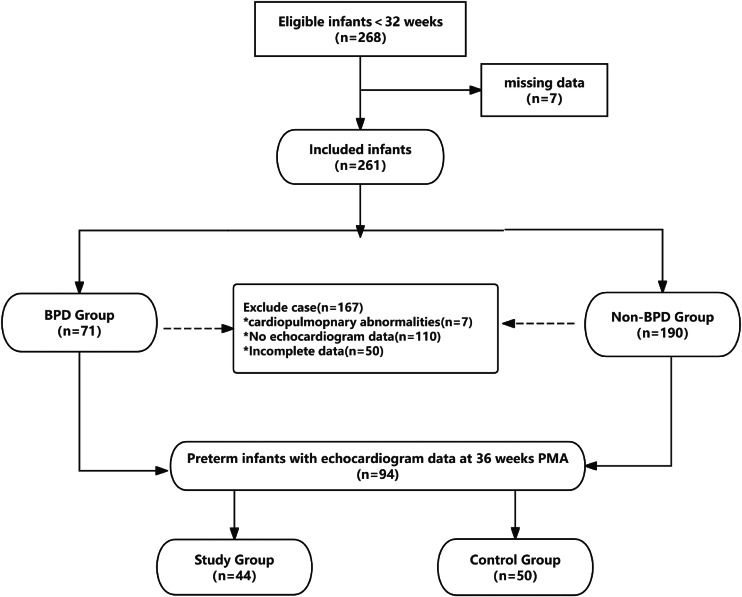
Recruitment flow chart.

From the original cohort of 268 infants, 94 were selected for this study: 50 without the diagnosis of BPD and 44 with BPD as assessed at 36 weeks of PMA (Figure 1). Patient characteristics are listed in Table 1. Infants with BPD exhibited significantly lower birth weight (1596 ± 106 g vs. 1731 ± 118 g, *p* < 0.01) and earlier gestational age at delivery (29.2 ± 0.67 weeks vs. 30.4 ± 0.64 weeks, *p* < 0.01) compared to the non-BPD control group. No significant differences were observed in the incidence of necrotizing enterocolitis (NEC), neonatal respiratory distress syndrome (NRDS), sepsis, pneumonia, pulmonary hypertension (pH), or intraventricular hemorrhage (IVH) (all *p* > 0.05).

**Table1:** Comparative analysis of clinical data between the two groups.

	Study Group (*n* = 44)	Control Group (*n* = 50)	*P-value*	OR(95 %CI)
Gestational age (week) mean (SD)	29.2 ± 0.67	30.4 ± 0.64	< 0.01	-
Birth weight (gram) mean (SD)	1596 ± 106	1731±118	< 0.01	-
Gender (male) [n( %)]	20 (45.5)	30(60)	0.16	0.55 (0.247–1.314)
Days of MV(day) mean (SD)	8.2 ± 2.3	3.5 ± 1.1	< 0.01	-
PDA [n( %)]	16(36.4)	15(30)	0.51	1.33 (0.554–3.236)
NRDS [n( %)]	12(27.3)	10(20)	0.40	1.5 (0.571–3.719)
NEC [n( %)]	4 (9.1)	3 (6)	0.70	1.56 (0.399–6.478)
Sepsis [n( %)]	9 (20.5)	13(26)	0.52	0.73 (0.291–1.94)
Pneumonia [n( %)]	4 (9.1)	3(6)	0.70	1.56 (0.399–6.478)
pH [n( %)]	3(6.8)	1(2)	0.33	3.58 (0.511–47.38)
IVH [n( %)]	3(6.8)	2(4)	0.66	1.75 (0.343–10.21)

MV, Mechanical ventilation; PDA, Patent ductus arteriosus; NRDS, Neonatal respiratory distress syndrome; NEC, Necrotizing enterocolitis; pH, Pulmonary hypertension; IVH, Intraventricular hemorrhage.

### Comparative analysis of right ventricular function between the two groups

Echocardiographic data are summarized in Table 2. Comparative analysis of right ventricular (RV) function indices revealed distinct differences between the study group and the control group. Specifically, no statistically significant differences were observed in right ventricular end-diastolic area (RVEDA) or right ventricular end-systolic area (RVESA) between the two groups (all *p* > 0.05). In contrast, the study group exhibited significantly altered values in two critical right ventricular functional parameters: right ventricular fractional area change (RV-FAC) and tricuspid annular plane systolic excursion (TAPSE) (all *p* < 0.05). Notably, tricuspid valve inflow velocities, including early diastolic inflow velocity (TV-E), late diastolic inflow velocity (TV-A), and the TV-E/A ratio, did not differ significantly between the groups (all *p* > 0.05; Table 2).

**Table 2:** Comparison of right ventricular function parameters between the two groups of infants.

	Study Group (*n* = 44)	Control Group (*n* = 50)	*P*-value
RVEDA (mm)	4.2 ± 1.7	3.8 ± 0.9	0.15
RVESA (mm)	2.8 ± 1.1	2.4 ± 1.1	0.08
FAC ( %)	33.2 ± 5.3	36.6 ± 6.3	0.01
TAPSE (mm)	6.4 ± 1.3	7.6 ± 1.4	< 0.01
TV-E (cm/s)	57.2 ± 15.67	52.2 ± 12.86	0.10
TV-A (cm/s)	56.3 ± 11.6	54.7 ± 6.9	0.41
TV-E/A ratio	1.0 ± 0.8	0.9 ± 0.5	0.47

Data are expressed as mean ±SD. RVEDA, right ventricular end-diastolic area; RVESA, right ventricular end-systolic area; FAC, fractional area change; TAPSE, tricuspid annular plane systolic excursion; TV-E, tricuspid E wave; TV-A, the tricuspid A wave; TV-E/A, the tricuspid E/A ratio.

### Comparative analysis of left ventricular function between the two groups

This study identified distinct patterns in left ventricular (LV) function between the BPD group and the control group. No statistically significant differences were detected in conventional LV structural and functional parameters, including left ventricular stroke volume (LVSV), left ventricular output (LVO), left ventricular fractional shortening (LVFS), left ventricular end-systolic dimension (LVESD), left ventricular end-diastolic dimension (LVEDD), and mitral valve late diastolic peak velocity (MV-A) (all *p* > 0.05). However, the BPD group demonstrated significantly impaired left ventricular diastolic function, which was evidenced by lower values of mitral valve early diastolic peak velocity (MV-E) and reduced MV-E/A ratio (both *p* < 0.05). Notably, a statistically significant intergroup difference was also observed in left ventricular ejection fraction (LVEF) (*p* < 0.05; Table 3).

**Table 3:** Comparison of left ventricular function parameters between the two groups of infants.

	Study Group (*n* = 44)	Control Group (*n* = 50)	*P*-value
LVSV	1.25 ± 0.2	1.29 ± 0.1	0.22
LVO (ml/min)	183 ± 47	191 ± 33	0.34
LVFS ( %)	33.2 ± 3.3	33.6 ± 2.4	0.50
LVESD (mm)	9.8 ± 1.2	10.1 ± 1.1	0.21
LVEDD (mm)	15.9 ± 1.6	16.2 ± 1.3	0.32
LVEF ( %)	67.8 ± 6.8	72.2 ± 7.3	< 0.01
MV-E (cm/s)	48.46 ± 10.4	65.24 ± 12.5	< 0.01
MV-A (cm/s)	70.34 ± 13.6	75.85 ± 14.7	0.06
MV-E/A ratio	0.58 ± 0.3	0.8 ± 0.4	< 0.01

Data are expressed as mean ±SD. LVSV, left ventricular stroke volume; LVO, left ventricular output; LVFS, left ventricular fractional shortening; LVESD, left ventricular end-systolic dimension; LVEDD, left ventricular end-diastolic dimension; LVEF, left ventricular ejection fraction; MV-E, the mitral E wave; MV-A, the mitral A wave; MV-E/A, the mitral E/A ratio.

### Comparative analysis of ventricular function using tissue doppler imaging between the two groups

Tissue Doppler imaging (TDI) analysis revealed distinct patterns between the two groups. Specifically, no statistically significant intergroup differences were observed in mitral peak systolic wave (MV-S′), mitral early diastolic wave (MV-E′), mitral end-diastolic wave (MV-A′), and the MV-E/A′ ratio (all *p* > 0.05). In contrast, tricuspid peak systolic wave (TV-S′), tricuspid early diastolic wave (TV-E′), tricuspid end diastolic wave (TV-A′), and the TV-E/A′ ratio were significantly impaired in the study group compared to the control group (all *p* < 0.05) (Table 4).

**Table 4:** Comparative analysis of ventricular function using tissue Doppler imaging between the two groups.

	Study Group (*n* = 44)	Control Group (*n* = 50)	*P*-value
MV-E′ (cm/s)	6.8 ± 2.2	6.4 ± 1.6	0.31
MV-A′ (cm/s)	6.4 ± 2.2	5.6 ± 1.8	0.06
MV-S′ (cm/s)	4.5 ± 1.1	4.3 ± 1.3	0.43
MV-E/E′ ratio	11.5 ± 2.3	10.6 ± 2.5	0.07
TV-E′ (cm/s)	7.2 ± 2.4	10.3 ± 2.7	< 0.01
TV-A′ (cm/s)	8.8 ± 1.2	10.4 ± 1.6	< 0.01
TV-S′ (cm/s)	6.2 ± 1.1	9.3 ± 1.3	< 0.01
TV-E/E′ ratio	11.3 ± 2.3	9.6 ± 2.1	< 0.01

Data are expressed as mean ±SD. MV-E′, mitral early diastolic wave; MV-A′, mitral end-diastolic wave; MV-S′, mitral peak systolic wave; TV-E′, tricuspid early diastolic wave; TV-A′, tricuspid end diastolic wave; TV-S′, tricuspid peak systolic wave.

## Discussion

This study systematically analyzed alterations in TNE parameters among pediatric patients with BPD. Comparative analysis identified statistically significant differences between BPD and non-BPD cohorts across key cardiac function indices: FAC, TAPSE, LVEF, MV-E, MV-E/A ratio, and tricuspid valve-related tissue Doppler imaging (TDI) parameters (TV-E′, TV-A′, TV-S′, and TV-E/E′).

BPD-related pulmonary vascular remodeling initiates a pathological cascade involving increased vascular tension, altered reactivity, vasoconstriction, and elevated pulmonary vascular resistance [14], leading to significantly elevated RV afterload. Chronic afterload elevation, coupled with hypoxic episodes, can progress to RV dysfunction, hypertrophy, and severe ventricular failure [15], Notably, early signs of BPD and respiratory insufficiency may emerge as early as postnatal day 7 [16]. A cohort study of 1735 preterm infants (23–30 weeks’ gestation) revealed a biphasic oxygen requirement pattern: initial decline from birth to day 7, followed by a resurgence in the second postnatal week [17]. Additionally, Czernik et al. [18] suggested that BPD-associated pulmonary vascular disease may impair RV function within 2 weeks of birth, though further research is needed for validation.

RV function assessment is crucial in TNE, particularly for pulmonary hypertension patients. The RV's complex crescent shape challenges geometric modeling, complicating assessment [19]. ASE Pediatric Quantification Guidelines [20] suggest standardized measurements: basal/mid-cavity end-diastolic diameters, end-diastolic longitudinal length, apical four-chamber end-systolic/end-diastolic planar areas, FAC, and TAPSE. These validated metrics are valuable in neonatal care. While right ventricular end-diastolic area (RVEDA) and end-systolic area (RVESA) showed no statistically significant differences (*p* > 0.05), the fractional area change (FAC) was significantly lower in the BPD group (*p* = 0.006). This suggests that FAC, as a functional ratio, may be a more sensitive indicator of global RV systolic performance than individual planar dimensions, which can be subject to significant inter-patient geometric variability and may be underpowered in smaller cohorts. Interestingly, while only 6.8 % of our BPD cohort met the formal criteria for pH, significant reductions in FAC and TAPSE were observed. This suggests that RV systolic impairment may precede the clinical diagnosis of overt pH, possibly reflecting “subclinical” elevation in pulmonary vascular resistance or impaired RV-pulmonary arterial coupling in the early stages of alveolar simplification [21]. Furthermore, our results were compared against established preterm neonatal normative ranges (e.g., TAPSE values of 6–10 mm) rather than adult thresholds, confirming a true functional deficit in the BPD group [[22], [23], [24]]. Our findings regarding reduced TAPSE and FAC in BPD infants align with those recently reported by Chakr et al. [25], who identified early echocardiographic markers of pulmonary vascular disease in at-risk preterm infants. This consistency suggests that RV systolic impairment is a robust feature of the BPD cardiopulmonary phenotype from the early stages through to 36 weeks PMA.

Our analysis showed no significant intergroup differences in tricuspid valve (TV) Doppler parameters (TV-E, TV-A, TV-E/A ratio; all *p* > 0.05). This aligns with their role in assessing diastolic filling, not systolic function. Neonatal cardiovascular physiology likely explains the limited clinical impact of RV diastolic dysfunction on these indices. TNE allows for thorough qualitative and quantitative assessment of global and regional LV systolic function. A prospective study found elevated LVO in the first two postnatal weeks may predict BPD in preterm infants [26], though the role of PDA in this link needs further study. In our research, BPD and non-BPD cohorts showed no significant differences in LVSV or LVO (*p* > 0.05). Among chamber geometry indices, LVFS and LVEF are key for LV function evaluation in pediatric critical care [27,28], While some studies show age-related LVFS increases in BPD infants [29], others report no LVEF differences between BPD and non-BPD groups [30]. Our study noted a significant decrease in LVEF in BPD infants (*p* = 0.003) despite comparable LVFS (*p* = 0.50). This discrepancy likely stems from the fact that LVFS is a linear measurement that assumes a prolate ellipsoidal geometry, which may be less accurate in BPD infants due to altered ventricular interdependence and septal flattening. LVEF, calculated via the biplane Simpson’s method, more accurately reflects global contractility by accounting for volume changes, though it remains sensitive to the increased afterload common in BPD.

MV-E and MV-A are crucial echocardiographic markers for assessing LV diastolic function. MV-E reflects the left atrial (LA)-LV pressure gradient during early diastole, influenced by LV relaxation rate and LA pressure changes. Our study showed significantly lower MV-E values in BPD infants than controls (*p* < 0.05), indicating impaired LV diastolic function. Physiologically, MV-E should exceed MV-A, but BPD infants had a markedly reduced MV-E/A ratio (0.58), well below the normal threshold of 1, suggesting significant diastolic dysfunction that may worsen BPD pathology. Fetal and preterm infant myocardium shows A-wave dominance, and our analysis found no significant intergroup difference in MV-A (*p* > 0.05). While an MV-E/A ratio < 1 indicates dysfunction in adults, preterm infants need a stricter threshold of < 0.6, and our BPD cohort's mean ratio (0.58 ± 0.3) meets this criterion. As a multifactorial disease, BPD's inflammatory pathways may increase ventricular afterload, LA pressure, and disrupt filling patterns [31]. Our findings align with previous studies linking LV diastolic impairment in BPD infants to prolonged diastolic intervals, altering the MV-E/A balance. The observed LV diastolic dysfunction (lower MV-E and MV-E/A ratio) suggests impaired myocardial relaxation. While systemic blood pressure was within normal ranges for both groups, the potential role of occult systemic hypertension or increased LV afterload from chronic inflammation cannot be ruled out as a contributor to altered filling patterns.

Tissue Doppler imaging (TDI) has emerged as a valuable tool for evaluating myocardial development in preterm infants. In our study, mitral annular TDI parameters—including MV-S′, MV-E′, MV-A′, and the MV-E/E′ — were comparable between BPD and non-BPD groups (all *p* > 0.05). By contrast, BPD infants exhibited significantly reduced tricuspid annular velocities (TV-S′, TV-E′, TV-A′) relative to controls (all *p* < 0.05), suggesting targeted RV dysfunction.

TV-S′ is a reliable marker of global RV systolic function. While adult studies define impaired RV systolic function as TV-S′ < 9 cm/s, normative data for preterm infants remain limited. Our findings align with prior research [28] which reported lower TV-S′ velocities in mild BPD infants at postnatal day 1 (6.2 ± 1.1 cm/s) and day 28 (9.3 ± 1.3 cm/s, *p* < 0.01) compared to non-BPD peers. Existing evidence indicates an age-dependent increase in TV-S′ during childhood, with neonates showing the most pronounced progression — likely reflecting concurrent cardiac growth and functional maturation. Furthermore, the significantly lower TV-S′ velocities observed in our BPD cohort are supported by the findings of Chakr et al. [25], further validating the utility of tricuspid annular dynamics as a sensitive indicator of targeted RV dysfunction in this population.

Among TDI parameters, the TV-E/E′ ratio is the most clinically validated measure of RV filling pressure. It directly reflects the hemodynamic consequences of diastolic dysfunction: elevated ventricular filling pressure manifests as a marked increase in TV-E/E′. Adult studies have established TV-E/E′ as a reliable correlate of elevated end-diastolic pressure and an independent predictor of heart failure. Supporting this, Akcan et al. [29] demonstrated via retrospective analysis that TV-E/E′ not only distinguishes BPD preterm infants from healthy controls but also quantifies BPD severity. Our data corroborate these observations, with statistically significant differences in TV-E/E′ ratios between BPD and control groups (*p* < 0.05).

This retrospective study has three limitations that should be considered when interpreting our findings. First, the relatively small size of our infant cohort may restrict statistical power, which could limit our ability to detect subtle intergroup differences; additionally, as is inherent to retrospective designs, our study is susceptible to selection bias and unmeasured confounders — such as variations in clinical management protocols not captured in medical records — that may have influenced the observed outcomes. Second, given that the study was conducted exclusively at a single tertiary care center over a 6-year period, the findings may be constrained by institutional-specific practices, including standardized TNE imaging protocols and approaches to comorbidity management; further, the authors were unable to fully account for comorbidities like pulmonary hypertension, which are known to affect cardiac function and could thus confound TNE parameter measurements. Third, our inclusion criteria were restricted to very low birth weight infants with available TNE data at 36 weeks PMA, which excluded infants with early BPD diagnoses; this focus on late-stage assessment may omit clinically relevant early-stage cardiac functional changes associated with BPD, ultimately limiting the generalizability of our results to only infants with established, late-stage BPD.

In conclusion, TNE is a viable noninvasive method for assessing BPD. Clinically, these findings emphasize that BPD management should transition from a purely “lung-centric” approach to a “cardiopulmonary” model. Early detection of biventricular dysfunction via TNE can guide precision interventions. Yet, prospective multicenter studies with standardized protocols are required to validate its efficacy in early BPD detection.

## Authors’ contributions

Conceptualization: Lie Huang, Jianhui Wang; Data curation: Meile Cheng, Lei Luo, Yongping Li; Formal analysis: Xi Xu; Resources: Meile Cheng; Writing – original draft: Xi Xu; Writing – review & editing: Lie Huang.

## Funding

None.

## Data availability

The datasets are not publicly available due to ethical restrictions and institutional privacy policies. The data contains sensitive information that could compromise the anonymity of the participants. Requests to access the data should be directed to the corresponding author.

## Conflicts of interest

The authors declare that they have no conflict of interest.

## Appendix Supplementary materials

Image, application 1

## Footnotes

Supplementary material associated with this article can be found in the online version at doi:10.1016/j.jped.2026.101521.
