# Supplementary material for: Clinical utility of echocardiography in bronchopulmonary dysplasia: a retrospective cohort study
Source: J Pediatr (Rio J). 2026 Mar 1;102(3):101521. doi: 10.1016/j.jped.2026.101521 (PMC12961200; doi:10.1016/j.jped.2026.101521)
Supplement: Supplementary file 1 [file mmc1.docx]

**JPED-D-25-00525_Supplementary Material**

**Table S1** Summary of Echocardiographic Parameters and Abbreviations.

| **Category** | **Echocardiographic Parameter** | **Abbreviation** | **Functional Significance** |
| --- | --- | --- | --- |
| LV Systolic Function | Left Ventricular Stroke Volume | LVSV | Volume of blood ejected per beat |
|  | Left Ventricular Output | LVO | Total volume of blood ejected per minute |
|  | LV Fractional Shortening | LVFS | Linear measurement of LV contractility |
|  | LV Ejection Fraction | LVEF | Volumetric measurement of LV contractility |
|  | LV End-systolic/End-diastolic Dimension | LVESD / LVEDD | LV chamber size at end-systole/diastole |
| RV Systolic Function | RV End-diastolic/End-systolic Area | RVEDA / RVESA | RV chamber size during cardiac cycle |
|  | RV Fractional Area Change | FAC | Percentage change in RV area |
|  | Tricuspid Annular Plane Systolic Excursion | TAPSE | Longitudinal displacement of the RV |
| Diastolic Function (PW) | Mitral Early/Late Diastolic Velocity | MV-E / MV-A | LV filling patterns (E/A ratio) |
|  | Tricuspid Early/Late Diastolic Velocity | TV-E / TV-A | RV filling patterns (E/A ratio) |
| Tissue Doppler (TDI) | Mitral/Tricuspid Systolic Peak Velocity | MV-S' / TV-S' | Peak annular velocity during systole |
|  | Mitral/Tricuspid Early Diastolic Velocity | MV-E' / TV-E' | Early diastolic myocardial relaxation |
|  | Mitral/Tricuspid Late Diastolic Velocity | MV-A' / TV-A' | Late diastolic myocardial relaxation |
|  | Mitral/Tricuspid E/E' Ratio | MV-E/E' / TV-E/E' | Indicator of ventricular filling pressures |
